# Bedside ultrasound diagnosis of a malpositioned central venous catheter: A case report

**DOI:** 10.1097/MD.0000000000010501

**Published:** 2018-04-13

**Authors:** Feier Song, Daozheng Huang, Ying Chen, Zhiyuan Xiao, Ke Su, Jianyi Wen, Weixin Guo, Zhonghua Wang, Yan Wu, Shouhong Wang, Tiehe Qin

**Affiliations:** a Department of Cardiology, Guangdong Cardiovascular Institue, Guangdong General Hospital, Guangdong Academy of Medical Sciences; b Department of Critical Care Medicine, Guangdong Geriatric Institute, Guangdong General Hospital, Guangdong Academy of Medical Sciences; c Department of Critical Care Medicine, Yunnan Cancer Hospital; d Department of Critical Care Medicine, Zhongshan Dongsheng Hospital, Guangzhou, Guangdong Province, China.

**Keywords:** bedside ultrasound, central venous catheter, malposition

## Abstract

**Rationale:**

Central venous catheter (CVC) placement is commonly performed in intensive care unit. And CVC placement is associated with risks including CVC malposition, pneumothorax. Many of the previously reported cases are about catheter misplacement detected by bedside ultrasound, chest x-ray (CXR) and computed tomography. In this case, malposition was detected by bedside ultrasound incidentally particularly with no clinical manifestation.

**Patient concerns:**

An 88-year-old male with severe diabetic peripheral neuropathy secondary to type 2 diabetes mellitus was admitted for further treatment.

**Diagnoses:**

We cannulated a single-lumen CVC via the right subclavian vein, and the tip ended up in the internal jugular vein on the same side. With bedside ultrasound, we discovered the malposition though it was mistaken by aspiration of venous blood. Later, CXR revealed malposition of the tip once again.

**Interventions:**

Since the patient was asymptomatic and the catheter was functioning normally, the catheter was used for the following 20 days without complications. Ultimately, we carefully performed the catheter removal.

**Outcomes:**

After the inserted catheter was removed, we attempted a new CVC through the left internal jugular vein. After the procedure, bedside ultrasound and CXR confirmed the correct position of CVC. Following successful replacement of the central catheter, no further complications were observed.

**Lessons:**

Bedside ultrasound offers safety and effectiveness during insertion of CVC. It also exhibits promptness and accuracy compared to post-intervention radiological imaging.

## Abstract 2

Supplemental Digital Content is available in the text

## 1 Introduction

Central venous catheter (CVC) placement is commonly performed in the management of critically ill patients.^[12]^ CVC functions in the delivery of intravenous medication and parenteral nutrition, transfusions, drawing blood for laboratory testing, and hemodynamic monitoring such as central venous pressure (CVP).

Percutaneous central catheter insertion is considered a safe but invasive procedure, as the mechanical complications vary from 3% to 19%.^[11,13,18]^ The optimal position for the CVC tip is the junction of the superior vena cava and right atrium, without contacting the pericardial reflection, to avoid cardiac perforation or tamponade and to reduce the risk of dysrhythmia. Besides, CVC placement is associated with risks, including CVC malposition, iatrogenic pneumothorax, arterial puncture and unrecognized arterial placement, infection, venous thrombosis, perforation or erosion of vessel walls, hemorrhage, and malfunction of the catheter.^[5,12,13]^ The incidence of CVC malposition is up to 14%.^[15]^ With potential catastrophic consequences, the CVC malposition requires additional monitoring and possible procedural interventions.

Although assistance such as ultrasound guidance decreases the rate of failing to locate correctly, considerable numbers of catheter malposition can still occur.^[16]^ Generally, catheter malposition occurs most commonly when a left internal jugular vein or subclavian vein is cannulated due to anatomic differences. Bedside ultrasound for confirmation of CVC position and exclusion of pneumothorax includes direct ultrasound identification of the aberrant placement within the internal jugular or subclavian vein as well as the typical ultrasonogram of pneumothorax such as lung sliding, lung point, B lines, and lung pulse.^[9]^

Here, we report a case of a critically ill patient in which a CVC tip was inadvertently inserted into the right internal jugular vein via the right subclavian vein, and its malposition was detected by bedside ultrasound. The bedside ultrasound showed its promptness and diagnostic accuracy for the confirmation of CVC position and identification of pneumothorax.

## 2 Case report

An 88-year-old male with severe diabetic peripheral neuropathy secondary to type 2 diabetes mellitus that had been diagnosed >20 years previous with complaints of exacerbation of dry mouth was admitted for further treatment. He was managed with blood glucose control by using insulin injections. However, a fever accompanied by increasing white blood cell and leucocyturia indicated an infection requiring antibiotics. A simple CXR revealed pneumonia and pleural effusion in both lower lung. Considering the aged patient also presented with edema with limbs and hypoproteinemia, a single-lumen CVC was introduced into the right subclavian vein to maintain robust and secure intravenous access, by an experienced physician.

The patient was placed in the supine position and the right subclavian vein was punctured, blood was easily aspirated, and the guide wire was inserted through the needle without any resistance up to 20 cm and the needle was removed. After dilating the skin and subcutaneous tissue, a one-lumen catheter was pushed in along the guide wire and inserted to a depth of 15 cm using the Seldinger technique. No resistances were felt during the initial insertion of both the guide and the catheter, and we confirmed aspiration of venous blood through the catheter. After insertion, the patient complained no obvious symptoms. Moreover, there was no noticeable resistance, arrhythmia, or changes in vital signs were observed during the initial insertion. No clinical manifestation was intensified by flushing the catheter with saline solution. Introduction of intravenous fluids resulted in corresponding clinical responses that further supported that the catheter was functioning.

**Figure 1:**
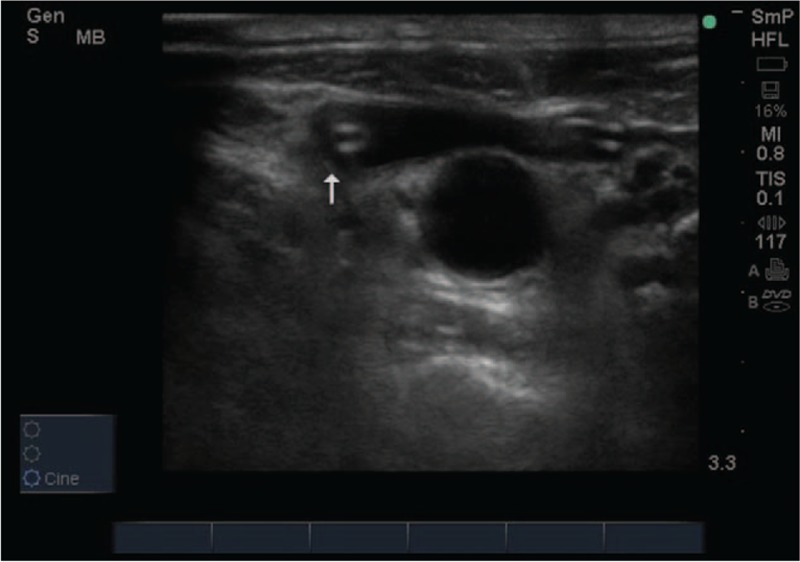
Ultrasonography also revealed two deep vein catheters in the internal jugular vein (two white arrow), indicating central venous catheter in the same side of right internal jugular and with it's tip making a U-turn downward.

After the procedure, we arranged the bedside ultrasound to rule out pneumothorax and other complications. Vascular ultrasound of the jugular vessels detected the immediate appearance of venous tube in the right internal jugular vein from 2 parallel hyperechoic lines consistent with a portion of a catheter, thus establishing that the kinking tube forming a U-turn shape, eventually led the tip close to the junction between internal jugular vein and brachiocephalic vein (Fig. 1, Supplementary Appendix 1). It was concluded that the catheter had entered the right internal jugular vein directly, but its tip remained downward without causing severe complications and somehow partially functioned. Color ultrasonic Doppler ruled out extravascular effusion or vessel perforation.

The posteroanterior chest radiograph was obtained to confirm the position of the catheter tip, showing it ascending into the right internal jugular vein. The CXR definitely disclosed catheter tip positioning in the right internal jugular vein, the radiologist also reported catheter malposition. No signs of pneumothorax were present, and the patient had no chest pain or dyspnea.

**Figure 2:**
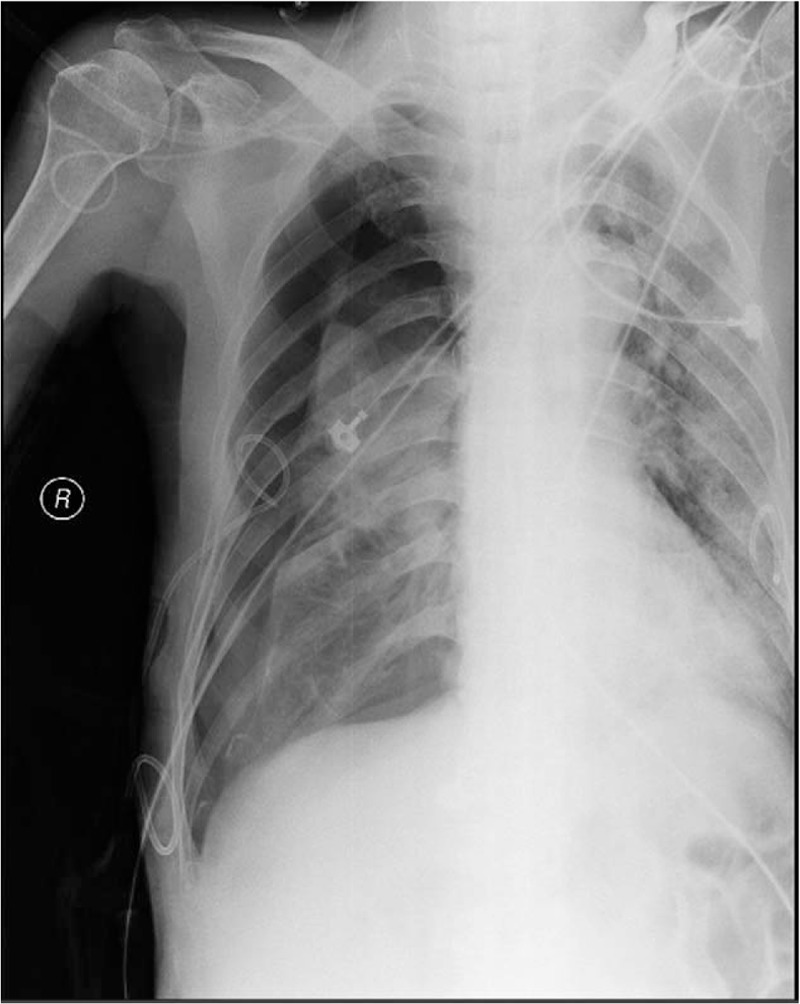
Anterior-posterior chest radiography shows the catheter into the right internal jugular vein, with its tip making a U-turn downward (red arrow show). The CXR also showed that the presence of right pneumothorax line (white arrow show) and confirmed pneumothorax.

**Figure 3:**
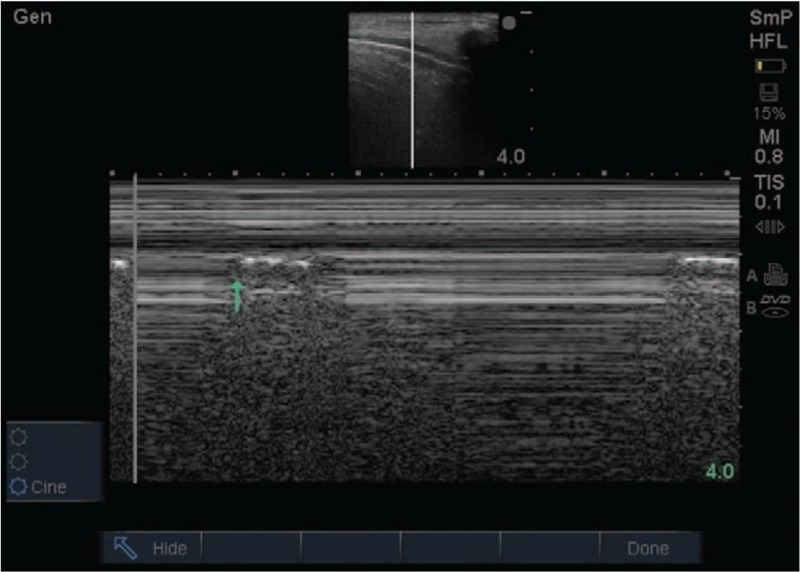
The presence of lung point (the green arrow show) in the M-mode can be used to diagnose pneumothorax in the bedside. The lung points in M-mode are characterized by a seashore sign and a stratospheric sign alternately.

The patient also had chest tube drainage on the right side. We next performed a bedside ultrasound at the anterior chest wall in the supine position and found out the presence of typical lung point (Fig. 3, Supplementary Appendix 2). In particular, abolished lung sliding, plus the A-line sign, which was a fundamental ultrasonographic sign highly specific for confirming pneumothorax. However, typical clinical manifestations such as dyspnea or decreasing of pulse oxygen saturation were still missing. Because some normal physiological conditions can be misdiagnosed for false lung points,^[20]^ we later perform the CXR to draw the conclusion (Fig. 2). Once again, the diagnosis of CXR was consistent with bedside ultrasound.

The malpositioned catheter should always be removed due to the risks such as embolization, perforation, and consecutive pericardial tamponade.^[19]^ In the present patient, the catheter functioned properly, and there were no apparent adverse effects from the malpositioned CVC. As the patient was asymptomatic and the catheter was functioning normally, the catheter was used for the following 20 days without complications. Ultimately, we carefully performed the catheter removal. After the inserted catheter was removed, we attempted a new CVC through the left internal jugular vein. This time, we applied the ultrasound-guiding technique, paid attention to the direction of the guide wire to guarantee the catheter was located in the right place. After the procedure, bedside ultrasound was performed once again to reassure the intravenous placement and did not require further treatment. A routine CXR was reported as showing a left-sided CVC correctly terminating in the superior vena cava. After successful replacement of the central catheter, no further complications were observed. Malposition of central venous cannulation via the subclavian vein is a rare and hazardous event.^[5]^ The risk for this complication is increased if catheters are inserted in left-sided veins.^[12]^

## 3 Discussion

We presented a case of CVC malposition through the subclavian vein pathway. One possible explanation is the wrong direction of the guide wire. When the guide wire enters the superior vena cava via the right subclavian vein, it forms a relatively acute downward bend, which may be wrongly placed and stop the sheath, expander, or CVC from the correct location.^[14]^ Because the catheter is placed inside the internal jugular vein, the aspiration of dark venous blood confused the correct positioning of the catheter. This may explain our misinterpretation of proper catheter placement. We speculate that in the initial insertion process, the guide wire entered the internal jugular vein, and the tip followed it right after. The process of catheter insertion seemed successful and CVP was not obtained because of the single-lumen catheter. Our patient had no apparent risk factors such as chronic obstructive pulmonary disease, obesity, cachexia, scoliosis, the abnormal anatomy of cardiothoracic structures.^[2]^ A study has demonstrated that the time required for catheter insertion and operator experience were relatively independent risk factors for complication rates.^[15]^ Despite the operator's expertise, the time pressures of the emergency setting could have influenced the risk of complication in this case.

Catheter malposition can occur as an immediate complication on insertion or rarely at a later stage. However, in our case, the catheter functioned properly, and there were no apparent adverse effects from the malpositioned CVC. The malposition was discovered incidentally during bedside ultrasound subsequently performed to rule out complications such as pneumothorax and hemorrhage.

In our case, bedside ultrasound showed that catheter ascending to the internal jugular vein distally, while the tip was turning down and running alongside the right brachiocephalic vein, which we suspected would allow the fluid to be easily infused in the central vein without resistance. Inadvertent placement of the CVC into the internal jugular vein is rare. In addition, placement of the CVC within the internal jugular vein can lead to subcutaneous bleeding, venous thrombosis, venous occlusion, and perforation. It is associated with serious morbidity and mortality unless the complications can be confirmed and correct immediately. Therefore, we often carry out bedside ultrasounds to detect complications such as pneumothorax and correct catheter placement as soon as possible.

Complications related to either catheter insertion or the presence of malposition are not quite infrequent. Thus, we emphasize the need for bedside ultrasound or radiographic confirmation for the sake of appropriate CVC placement, no matter how smoothly the insertion proceeded particularly after a blind procedure with some unsuccessful attempts.

In the last few years, CXR were routinely performed after CVC implants to identify postoperative complications, such as pneumothorax, and to confirm the proper placement of the catheter.^[17]^ In our case, postoperative ultrasonography was suspicious of catheter malposition. CXR also indicates the abnormal position of the catheter tip, but it was interpreted to possibly represent the catheter terminating nearly in the right brachiocephalic vein, which might be considered hemicentral vein, and no further action was taken.

Bedside ultrasound has advantages over chest radiography including lack of ionizing radiation exposure, decreased resource utilization, and decreased diagnostic time. This earlier confirmation of CVC positioning allows for earlier clinical management which could potentially affect patient outcomes. Bedside ultrasound relies greatly on the experience of the operator; it is advocated for routine postprocedural surveillance of CVC placement for its time efficiency and relative accuracy. Moreover, we recommend the diagnosis be rechecked by X-ray especially when the symptoms are ambiguous.

In our case, the malposition of the catheter and lung point was discovered accidentally by the bedside ultrasound. In retrospect, they could be a process-related complication. The exact location of CVC was concluded by CXR. However, the normal function of CVC, as well as the absence of any obvious complications due to CVC placement, may be the reason for failure to realize the malposition of CVC. By ultrasound, we have not only identified the tip of the catheter but also excluded hematoma or peripheral tissue hemorrhage.

It is suggested that the tip of a CVC should be at the junction of the superior vena cava and right atrium. CXR is the usual modality used to evaluate proper catheter position and detect pneumothorax, but it cannot always be timely and feasible in critical ill settings, and in such instances, bedside ultrasound may be a prompter and more accurate means of confirming the position of the CVC tip, as was the situation in our case. One could speculate that with optimal images, ultrasound might have provided similar information to x-ray. Bedside ultrasound correctly identified the location of the catheter tip and pneumothorax through lung point,^[9]^ and radiologic imaging confirmed it and established the direct diagnosis of pneumothorax offering the evaluation of severity. Our case shows that bedside ultrasound accurately identifies malposition and pneumothorax after CVC insertion. Importantly, ultrasound provides results regarding catheter position and pneumothorax faster than chest radiography.

Considering the conclusions of previous research, we recommend that bedside ultrasound is used as first-line confirmation method to determine catheter malposition. If the CVC is found to be malpositioned in a venous structure, the CVC can be expediently addressed without obtaining a chest radiograph first. However, sometimes it is skeptical of CVC malposition but is not detected by ultrasound, especially in the case of multiple cannulation attempts, resistance during insertion, poor blood drawing, excessively high CVP. In this circumstance, chest radiography should be performed to rule out catheter malposition.

Ultrasound-guided insertion offers improved safety with significantly lower rates of mechanical complications. This case highlights that during the management of critically ill patients, despite safer methods of catheter insertion, catheter misplacement can still occur. It is unreliable to simply infer the correct placement through blood aspiration alone. Correct positioning of intravascular lines must be regularly and systematically checked before their use, as these may have been incorrectly sited initially or later displaced during hospital transfers.

In particular, in patients with risk factors, it is difficult to detect the malposition of the catheter tip during a blind procedure. Therefore, using image guidance such as ultrasonography might be crucial for identifying malposition. For approaches without ultrasound-guided process, an immediate postprocedural bedside check is essential. In addition, given the high incidence of bedside ultrasound use during the insertion of CVC, the efficiency of postinsertion confirmation should be augmented too.^[1]^

Numerous studies, review, and meta-analysis have demonstrated that bedside ultrasound can detect nearly all pneumothoraxes^[3,7,8,10]^ and 4 out of 5 catheter malpositions after CVC placement.^[1]^ It also shows that bedside cardiac ultrasound and lung ultrasound are feasible in >95% of patients and can be completed more efficiently than chest radiography. This meta-analysis shows that obtaining adequate ultrasound images for confirmation of CVC placement is necessary and has good interobserver reliability. However, clinician's ability and experience levels with ultrasound are still highly variable in actual practice.^[4]^

In our case, bedside ultrasound for CVC confirmation was associated with time reduction compared with chest radiography completion and chest radiography report as well. Bedside ultrasound offers safety and effectiveness during insertion of CVC.^[6]^ It also exhibits promptness and accuracy compared with aspirating blood and postintervention radiological imaging. We describe a rare complication of subclavian vein catheterization. Late complications can occur due to improper initial insertion or repeated puncture. Safe methods of catheter insertion, confirmation of correct placement, and continuous awareness of the possibility of procedure-related complications are the cornerstone for reducing adverse events.

## Acknowledgments

Ethical approval was not necessary. This is a retrospective, observative case report. All the invasive operation and treatment was approved by the patient and his families, with written informed consent. We obtained informed consent for publication of this case from the patient and the patient's name, ID and other private information will never disclose under any circumstance. The authors vouch for the accuracy and completeness of the data, as well as for the fidelity of this case report. The first author drafted the manuscript, and all the authors made the decision to submit the manuscript for publication.

## Author contributions

**Conceptualization:** Feier Song, Daozheng Huang.

**Data curation:** Ying Chen, Zhiyuan Xiao, Ke Su, Jianyi Wen, Weixin Guo, Zhonghua Wang.

**Investigation:** Feier Song, Shouhong Wang.

**Project administration:** Shouhong Wang, Tiehe Qin.

**Resources:** Yan Wu, Shouhong Wang.

**Writing—original draft:** Feier Song.

**Writing—review and editing:** Feier Song, Daozheng Huang, Ying Chen, Zhiyuan Xiao, Ke Su, Jianyi Wen, Weixin Guo, Zhonghua Wang, Yan Wu, Shouhong Wang, Tiehe Qin.

## Supplementary Material

Supplemental Digital Content

## Supplementary Material

Supplemental Digital Content
